# Structured-to-text ClinicalBERT embeddings with random Forest for heart disease prediction: a proof-of-concept study on the UCI Statlog dataset

**DOI:** 10.3389/frai.2026.1844707

**Published:** 2026-07-03

**Authors:** U. Priyadharshini, R. Vijayan

**Affiliations:** 1 School of Advanced Sciences, Vellore Institute of Technology (VIT), Vellore, India; 2 School of Computer Science Engineering and Information Systems, Vellore Institute of Technology (VIT), Vellore, India

**Keywords:** ClinicalBERT, clinical decision support, heart disease prediction, Natural Language Processing, Random Forest, structured-to-text conversion

## Abstract

Heart disease remains one of the leading causes of mortality worldwide, highlighting the need for accurate and early risk prediction systems. Traditional machine learning approaches for cardiovascular disease prediction primarily rely on structured clinical attributes and may not fully capture contextual relationships among patient features. To address this limitation, this study proposes a structured-to-text ClinicalBERT framework that transforms structured cardiovascular records into contextual clinical text representations and utilizes transformer-based embeddings for heart disease prediction. The study employs a publicly available UCI Statlog/Kaggle heart disease dataset containing 270 complete patient records. Structured cardiovascular attributes, including age, sex, chest pain type, blood pressure, cholesterol level, electrocardiogram results, and heart rate measurements, are converted into clinically meaningful textual descriptions. These text representations are processed using ClinicalBERT to generate contextual embeddings, which are subsequently used as input features for a Random Forest classifier. Model performance was evaluated using an 80:20 train-test split and assessed through Accuracy, Precision, Recall, F1-score, and ROC-AUC metrics. Experimental results demonstrate that the proposed ClinicalBERT + Random Forest framework achieved an accuracy of 95.6%, precision of 88.89%, recall of 95.30%, F1-score of 91.30%, and a ROC-AUC of 0.71 on the held-out test set. Comparative analysis with conventional machine learning models indicates that contextual embeddings generated by ClinicalBERT provide improved feature representation for cardiovascular risk prediction. The findings demonstrate the feasibility of adapting ClinicalBERT to structured cardiovascular data through contextual text generation. Although the proposed framework shows promising predictive performance, the study should be considered a proof-of-concept due to the limited dataset size and absence of external validation. Future work will focus on multicenter evaluation, explainable AI techniques, and broader clinical validation to enhance generalizability and real-world applicability.

## 1 Introduction

Cardiovascular disease Cardiovascular disease (CVD) remains one of the leading causes of mortality worldwide and continues to impose a significant burden on healthcare systems. Early identification of individuals at risk of heart disease is essential for timely intervention, improved patient outcomes, and reduced healthcare costs. Recent advances in Artificial Intelligence (AI), Machine Learning (ML), and Deep Learning (DL) have enabled the development of automated clinical decision-support systems capable of assisting clinicians in disease diagnosis and risk prediction. Traditional machine learning algorithms such as Logistic Regression, Support Vector Machine (SVM), Random Forest, and XGBoost have demonstrated promising performance for heart disease prediction using structured clinical attributes including age, blood pressure, cholesterol level, electrocardiogram findings, and heart rate measurements. However, these approaches generally treat each feature independently and may not effectively capture contextual relationships among patient attributes. Transformer-based language models have recently transformed Natural Language Processing (NLP) by learning contextual representations from textual data. Among these models, ClinicalBERT has gained considerable attention in healthcare applications because it is further pre-trained on clinical notes, electronic health records (EHRs), and biomedical text. As a result, ClinicalBERT can better understand medical terminology, abbreviations, and contextual clinical relationships compared with general-domain language models. ClinicalBERT has been successfully applied to clinical text classification, disease prediction, medical concept extraction, and healthcare decision-support systems.

Despite these advantages, the application of ClinicalBERT to structured cardiovascular datasets remains relatively unexplored. Most existing heart disease prediction studies rely on conventional machine learning methods directly applied to tabular data, while transformer-based models are typically designed for unstructured clinical narratives. Consequently, an important research gap exists in determining whether structured cardiovascular attributes can be transformed into meaningful clinical text representations that allow ClinicalBERT to extract richer contextual information than conventional tabular learning approaches. To address this challenge, this study introduces a structured-to-text adaptation strategy in which structured cardiovascular variables are converted into clinically meaningful sentences describing a patient’s condition. Rather than treating age, cholesterol level, blood pressure, ECG results, and chest pain characteristics as isolated variables, the proposed framework presents them in a contextual clinical format. This enables ClinicalBERT to model semantic relationships among cardiovascular risk factors and generate contextual embeddings that may better represent patient health status.

The generated ClinicalBERT embeddings are subsequently used as input features for a Random Forest classifier. Random Forest was selected because of its robustness on small and medium-sized healthcare datasets, ability to handle high-dimensional feature representations, resistance to overfitting, and strong performance in medical prediction tasks. Unlike end-to-end transformer fine-tuning, which requires larger datasets and substantial computational resources, the ClinicalBERT + Random Forest framework offers a computationally efficient alternative suitable for moderate-sized cardiovascular datasets. Preliminary experiments were also conducted using conventional machine learning models; however, Random Forest demonstrated the most stable performance when combined with ClinicalBERT embeddings, leading to its selection as the final classification model.

The proposed approach provides several advantages. First, it enables the utilization of ClinicalBERT’s contextual understanding capabilities on structured cardiovascular data through a structured-to-text conversion process. Second, it combines transformer-based feature extraction with a robust machine learning classifier, reducing computational complexity compared with fully fine-tuned deep learning architectures. Third, the framework can be implemented using publicly available healthcare datasets without requiring extensive clinical narratives. Nevertheless, certain limitations must be acknowledged. The generated clinical sentences are template-based representations and may not fully capture the complexity of real-world physician notes. Furthermore, the study relies on a publicly available cardiovascular dataset and does not include external multicenter validation. Therefore, the findings should be interpreted as a proof-of-concept investigation of structured-to-text ClinicalBERT adaptation for cardiovascular risk prediction. The main objective of this study is to evaluate whether contextual embeddings generated from structured cardiovascular records using ClinicalBERT can improve heart disease prediction performance. Specifically, the study aims to: (1) transform structured cardiovascular attributes into clinically meaningful textual descriptions; (2) generate contextual patient representations using ClinicalBERT embeddings; (3) classify heart disease risk using a Random Forest classifier; and (4) compare the predictive performance of the proposed framework with conventional machine learning approaches using standard evaluation metrics including Accuracy, Precision, Recall, F1-score, and ROC-AUC.

## 2 Literature review

### 2.1 Structured-to-text ClinicalBERT embeddings with Random Forest for heart disease prediction

Cardiovascular disease (CVD) remains a leading cause of mortality worldwide, motivating the development of intelligent prediction systems. Traditional machine learning models achieve good performance on structured cardiovascular data but often fail to capture contextual relationships among clinical variables. Recent transformer-based models such as ClinicalBERT have demonstrated strong representation learning capabilities in healthcare. However, their application to structured cardiovascular datasets remains limited. Therefore, this study investigates a structured-to-text transformation approach that enables ClinicalBERT to generate contextual embeddings from cardiovascular records, which are subsequently classified using a Random Forest model for heart disease prediction on the UCI Statlog dataset. ClinicalBERT was developed by pretraining BERT on clinical notes and electronic health records (Huang et al., 2019). The model demonstrated superior performance in clinical prediction tasks by capturing contextual medical information more effectively than general-purpose language models. This study introduced publicly available ClinicalBERT embeddings trained on clinical text data (Alsentzer et al., 2019). Results showed significant improvements in healthcare NLP tasks through domain-specific contextual representation learning. BEHRT applied transformer architectures to structured EHR data for disease prediction and patient representation learning (Li et al., 2020). The model effectively captured longitudinal healthcare information and outperformed conventional machine learning approaches. Med-BERT leveraged large-scale structured EHR data to generate contextual patient embeddings (Rasmy et al., 2021). The framework improved disease prediction accuracy by learning complex relationships among clinical variables. TabNet introduced an attention-based deep learning framework specifically designed for tabular datasets (Arik and Pfister, 2021). It achieved competitive prediction performance while providing improved model interpretability. TabTransformer applied transformer-based contextual embeddings to structured tabular data (Huang et al., 2020). The study demonstrated improved feature interaction modeling and classification accuracy compared with traditional methods. TaBERT jointly learned representations from both tables and text using transformer architectures (Yin et al., 2020). The model improved semantic understanding by linking structured attributes with textual context. TABLEFORMER introduced a transformer-based framework for robust table-text encoding, enabling effective representation learning from structured tabular information (Yang et al., 2022). The study demonstrated that transformer architectures can capture relationships between table contents and textual information, improving performance on table-related language understanding tasks. Domain-specific pretraining on biomedical and clinical text significantly improves language model performance in healthcare NLP tasks (Gu et al., 2021). The study demonstrates the effectiveness of contextual embeddings for clinical prediction and information extraction. Si et al. (2019) enhanced clinical concept extraction using contextual transformer embeddings trained on medical text. Results showed improved recognition of medical entities and semantic understanding in clinical narratives. This study employed a self-attention transformer architecture to predict cardiovascular disease risk (Rahman et al., 2023). Results indicated that transformer models effectively captured dependencies among heart disease risk factors. Johnson et al. (2016) developed transformer-based embeddings from electronic health records for clinical prediction. The framework achieved improved predictive performance by learning contextual patient representations. This review summarized recent advancements in AI, machine learning, and deep learning for cardiovascular diagnosis and prognosis (Krittanawong et al., 2019). The authors highlighted the growing role of transformer models in precision cardiology. Reviewed the role of Explainable Artificial Intelligence (XAI) in Healthcare 5.0, highlighting techniques such as SHAP, LIME, and attention-based explanations to improve transparency and trust in AI-driven clinical decision-making (Saraswat et al., 2022). The study also discussed key challenges, including interpretability, fairness, privacy, and regulatory compliance, emphasizing the need for explainable and clinically reliable healthcare AI systems. Pavlov et al. (2024) discussed the growing shift from traditional statistical methods toward machine learning techniques in cardiovascular pharmacotherapy, highlighting their ability to model complex and nonlinear clinical relationships. The study emphasized that while machine learning can improve predictive performance, clinical interpretability and plausibility remain essential for trustworthy decision-support systems.

### 2.2 Heart disease prediction, ClinicalBERT, EHRs, structured-to-text learning, transformers, and explainable AI

RETAIN introduced an interpretable attention mechanism for healthcare prediction (Choi et al., 2016). The model provides clinically meaningful explanations while maintaining high predictive accuracy. This study demonstrated large-scale deep learning on EHR data for disease prediction (Rajkomar et al., 2018). Results showed that deep neural networks can outperform traditional clinical prediction methods. Deep Patient learned patient representations from EHR data without manual feature engineering (Miotto et al., 2016). The framework improved prediction across multiple diseases. This landmark paper introduced the transformer architecture and self-attention mechanism (Vaswani et al., 2017). It forms the foundation for BERT, ClinicalBERT, and modern healthcare transformers. BERT introduced contextual language representation learning using bidirectional transformers (Devlin et al., 2019). It serves as the foundation for ClinicalBERT and other domain-specific language models. BioBERT adapted BERT for biomedical literature and clinical text processing (Lee et al., 2020). The model significantly improved healthcare NLP performance. Evaluated BERT and ELMo through transfer learning on ten biomedical NLP benchmark datasets and demonstrated that pretrained language models significantly improve performance across a variety of biomedical text-mining tasks (Peng et al., 2019). The study highlighted the effectiveness of contextual embeddings in capturing domain-specific biomedical knowledge and established BERT as a strong baseline for biomedical NLP applications. This survey reviewed deep learning methods applied to EHR analysis (Shickel et al., 2018). It highlighted challenges and opportunities in healthcare prediction systems. The paper introduced advanced SHAP-based explainability methods for machine learning models (Lundberg and Lee, 2017). These techniques improve transparency and trust in clinical decision-support systems. This study discussed how AI can augment clinical decision-making and precision medicine (Topol, 2019). It emphasized the importance of combining predictive accuracy with interpretability in healthcare. MedCAT extracts clinical concepts from healthcare records and transforms medical text into structured representations, supporting contextual clinical prediction (Kraljevic et al., 2021). This review compared deep learning architectures for Electronic Health Records and highlighted their ability to model complex patient data (Solares et al., 2020). The study emphasized the growing role of transformer-based methods in healthcare prediction. The study applied deep learning models to predict heart failure onset from longitudinal patient records (Choi et al., 2017). Results demonstrated improved early disease detection through temporal healthcare data modeling. Zhang et al. (2020) combined structured clinical variables with unstructured text to improve healthcare prediction accuracy. The framework demonstrated that integrating multiple data modalities enhances predictive performance. This study introduced SHAP-based explainable machine learning techniques for interpreting clinical predictions (Lundberg et al., 2018). The approach improved transparency by identifying the contribution of individual features to model decisions. Zeljkovic et al. (2025) evaluated GPT-4 for automated 12-lead ECG interpretation and found that providing relevant clinical context significantly influenced diagnostic accuracy and decision-making performance. The study demonstrated that AI diagnostic systems benefit from contextual clinical information, highlighting the importance of incorporating meaningful patient context rather than relying solely on isolated physiological data.

### 2.3 Structured-to-text ClinicalBERT framework for cardiovascular risk prediction

**Figure 1:**
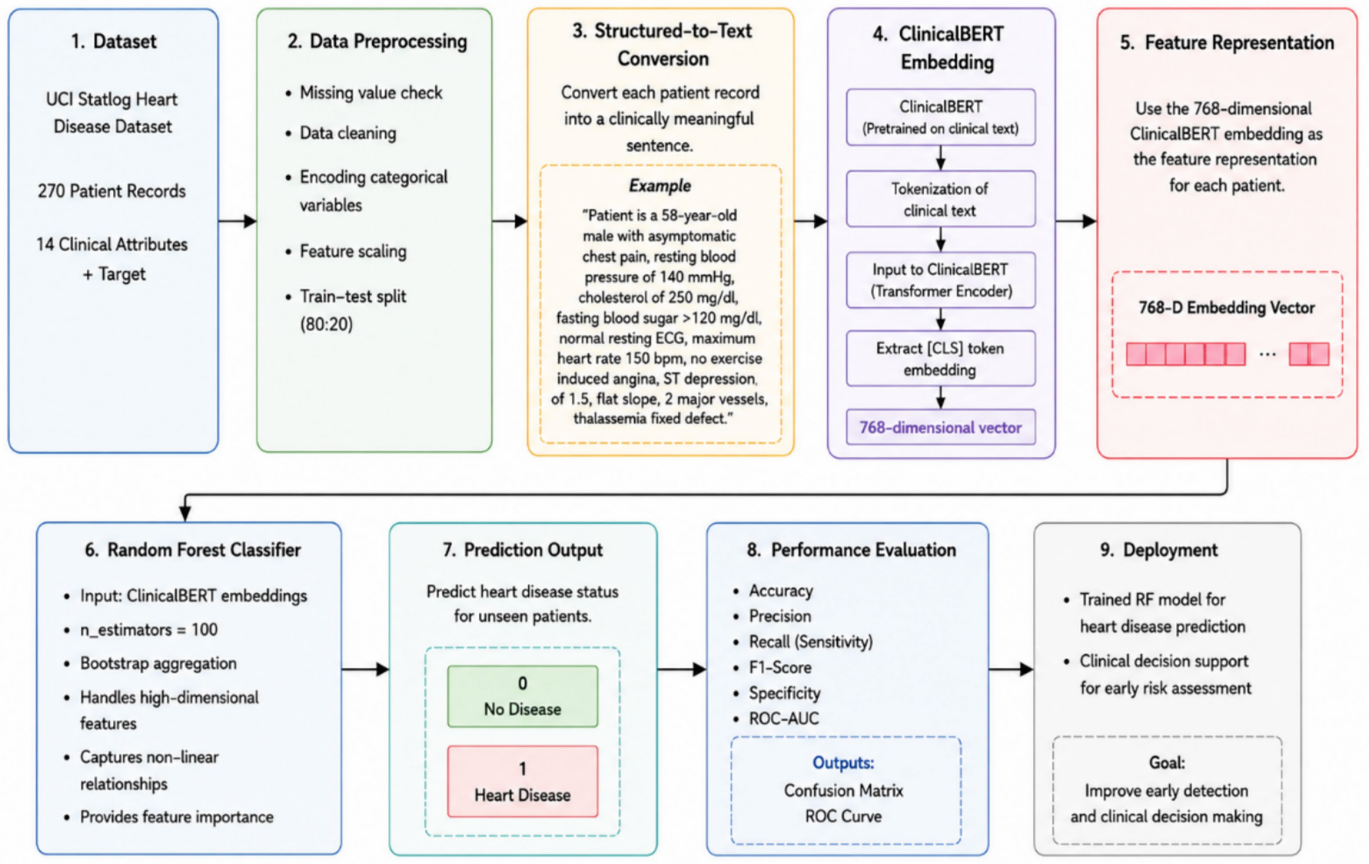
Architecture of the proposed ClinicalBERT–Random Forest heart disease prediction system.

ClinicalBERT was selected in this study because it is a domain-specific transformer model pretrained on large-scale clinical notes and electronic health records, enabling it to capture meaningful medical context and semantic relationships among clinical concepts. Since the UCI Statlog heart disease dataset contains structured cardiovascular attributes rather than free-text clinical narratives, a structured-to-text transformation strategy was adopted to convert each patient record into a clinically meaningful sentence. This approach allows ClinicalBERT to process cardiovascular information in a format similar to its pretraining data and generate contextual embeddings that represent interactions among multiple risk factors rather than treating them as isolated variables. The extracted ClinicalBERT embeddings were subsequently classified using a Random Forest model. Random Forest was chosen because the dataset contains only 270 records, making deep neural classifiers more susceptible to overfitting. Furthermore, Random Forest is effective for high-dimensional feature representations, captures nonlinear relationships, requires limited hyperparameter tuning, and provides robust performance on small datasets. Although other classifiers such as Logistic Regression, Support Vector Machines, and deep neural networks could be combined with ClinicalBERT embeddings, they were not adopted because Logistic Regression may not fully exploit complex feature interactions, Support Vector Machines can be computationally demanding for high-dimensional embeddings, and neural network classifiers generally require larger datasets for reliable generalization. Therefore, the ClinicalBERT + Random Forest framework was selected as a balanced approach that combines contextual representation learning with a robust and computationally efficient classifier for heart disease prediction. Figure 1 shows the proposed framework.

This study proposes a Structured-to-Text ClinicalBERT + Random Forest framework for heart disease prediction using the UCI Statlog Heart Disease dataset (270 records). The dataset was divided into 80% training (216 records) and 20% testing (54 records) using a fixed random seed to ensure reproducibility. Since ClinicalBERT is designed for clinical text rather than structured tabular data, each patient record was converted into a standardized clinical sentence containing cardiovascular attributes such as age, chest pain type, blood pressure, cholesterol level, ECG findings, and heart rate. The target label was excluded from the generated text to prevent data leakage. The generated clinical sentences were processed using Bio_ClinicalBERT, and the [CLS] token embedding was extracted as a 768-dimensional contextual representation of each patient record. ClinicalBERT was used as a frozen feature extractor without fine-tuning. The extracted embeddings were classified using a Random Forest model. Random Forest was selected because it performs effectively on small datasets, captures nonlinear relationships, reduces overfitting, and efficiently handles high-dimensional embedding features. This combination enables contextual feature learning through ClinicalBERT while maintaining robust classification performance. The proposed model was evaluated using Accuracy, Precision, Recall, Specificity, F1-Score, and ROC-AUC. The overall workflow consists of data preprocessing, structured-to-text conversion, ClinicalBERT embedding extraction, Random Forest classification, and performance evaluation. This proof-of-concept study investigates whether contextual embeddings derived from structured cardiovascular records can improve heart disease prediction.

## 3 Methodology

This section describes the proposed ClinicalBERT-based heart disease prediction framework, including dataset preparation, preprocessing, structured-to-text transformation, embedding generation, classification model, and evaluation metrics.

### 3.1 Dataset description

The study utilizes the publicly available UCI Heart Disease dataset, a widely used benchmark dataset in cardiovascular disease prediction research. It contains clinical and demographic attributes that are commonly used for diagnosing heart-related conditions. Table 1 represents the dataset features.

**Table 1:** Dataset attributes used for structured-to-text ClinicalBERT encoding.

Attribute	Type	Role in the revised pipeline
Age	Numerical	Demographic risk factor included directly in the generated clinical sentence.
Sex	Categorical	Encoded as a demographic descriptor and represented textually.
Chest pain type	Categorical	Symptom descriptor; encoded textually after categorical mapping.
Resting blood pressure	Numerical	Clinical measurement included as a numeric phrase in the generated sentence.
Serum cholesterol	Numerical	Biochemical cardiovascular risk indicator included as a numeric phrase.
Fasting blood sugar	Binary/categorical	Metabolic-risk indicator represented as present/absent or threshold-based text.
Resting ECG result	Categorical	ECG-derived diagnostic descriptor converted into textual form.
Maximum heart rate	Numerical	Exercise-response indicator included as a numeric clinical phrase.
Exercise-induced angina	Binary/categorical	Symptom indicator represented textually.
ST depression (oldpeak)	Numerical	ECG stress-test indicator retained as a continuous numeric descriptor.
Slope of ST segment	Categorical	ECG morphology descriptor converted into a clinical phrase.
Number of major vessels	Numerical/categorical	Angiographic descriptor included as a structured risk indicator.
Thalassemia status	Categorical	Diagnostic descriptor represented textually after category mapping.

### 3.2 Data preprocessing

Data preprocessing is performed to improve data quality and enhance model performance. The following steps are applied:

- Handling missing values: Incomplete records are removed or imputed to maintain dataset consistency.
- Feature normalization: Numerical attributes are scaled to a uniform range to prevent bias toward high-magnitude features.
- Removal of inconsistent entries: Noisy or contradictory data instances are filtered out.
- Encoding categorical variables: Non-numeric features such as chest pain type and ECG results are transformed into numerical representations using encoding techniques.

### 3.3 Implementation details and reproducibility

The proposed framework was implemented using Python 3.10, PyTorch 2.1, Transformers 4.38, Scikit-learn 1.4, and NumPy 1.26. Clinical text embeddings were generated using the Bio_ClinicalBERT checkpoint (“emilyalsentzer/Bio_ClinicalBERT”) with the corresponding WordPiece tokenizer. The maximum sequence length was set to 128 tokens. ClinicalBERT was used as a frozen feature extractor without fine-tuning, and the final [CLS] token representation (768 dimensions) was extracted as the patient embedding.

The dataset was divided using an 80:20 train-test split with a fixed random seed of 42, resulting in 216 training records and 54 testing records. The Random Forest classifier was configured with 200 estimators, maximum depth of 10, minimum samples split of 2, minimum samples leaf of 1, and random state of 42. Experiments were conducted on a system equipped with an Intel Core i7 processor, 16 GB RAM, and NVIDIA RTX-series GPU.

### 3.4 Structured-to-text conversion

#### 3.4.1 Example structured-to-text transformation

To ensure transparency and reproducibility, an example of the structured-to-text conversion process is provided below. Table 2 shows the original record.

**Table 2:** Original structured record.

Feature	Value
Age	58
Sex	Male
Chest pain type	Typical Angina
Resting BP	140 mmHg
Cholesterol	240 mg/dL
Exercise-induced angina	Yes

#### 3.4.2 Generated clinical sentence

**Figure 2:**
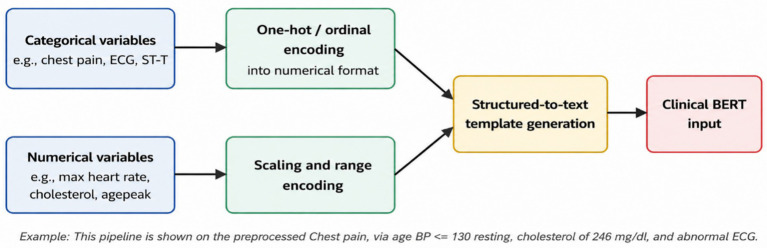
Feature encoding structured-to-text conversion.

“Patient is a 58-year-old male presenting with typical angina, resting blood pressure of 140 mmHg, serum cholesterol level of 240 mg/dL, and exercise-induced angina present.” Figure 2 shows the Feature encoding structured-to-text conversion.

The heart disease outcome label was never included in the generated sentence, embedding generation process, feature engineering stage, model selection procedure, or preprocessing pipeline, thereby preventing any possibility of label leakage.

### 3.5 ClinicalBERT feature extraction

Structured clinical patient records are first transformed into natural language clinical sentences to enable contextual understanding using transformer-based models. Each patient record is reformulated into a descriptive statement containing demographic information, symptoms, and clinical measurements (e.g., age, chest pain type, cholesterol level, and blood pressure). These sentences are then tokenized and processed using ClinicalBERT, a domain-specific variant of BERT pretrained on biomedical and clinical corpora. ClinicalBERT converts the tokenized input into dense vector representations through multiple transformer encoder layers. Within these layers, the self-attention mechanism learns contextual dependencies between clinical attributes by assigning dynamic importance weights to different tokens, enabling the model to capture relationships between symptoms and disease risk factors. Multi-head attention further enhances representation learning by focusing on different aspects of the clinical data simultaneously, such as demographic factors, symptoms, and physiological measurements. The resulting output is a set of contextualized embeddings, typically represented by the [CLS] token embedding, which encodes the overall semantic meaning of the patient record. These high-dimensional embeddings effectively preserve complex inter-feature relationships and are subsequently utilized as input features for the Random Forest classifier to perform heart disease prediction.

### 3.6 Random Forest classification

Random Forest is used as the final classification layer due to its strong performance in handling complex medical data. It is a robust ensemble method that reduces the impact of noise and outliers by combining multiple decision trees. This approach helps to minimize overfitting and improves the model’s ability to generalize well on unseen data. Additionally, Random Forest is effective in capturing nonlinear relationships between clinical features, making it highly suitable for accurate heart disease prediction. ClinicalBERT + Random Forest Heart Disease Prediction Pipeline Algorithm given in Algorithm 1.

### 3.7 Prediction model and evaluation equations

Let *D* = {(*x_i_, y_i_*)}*^N^* denote the patient-level dataset, where *x_i_* is the vector of structured cardiovascular attributes and *y_i_* ∈ {0, 1} is the binary heart disease label. The structured cardiovascular attributes were transformed into textual representations using the mapping function described in Equation 1.


$$\;s_{i}=g(x_{i}),\;\;i=1,2,\ldots ,N$$ (1)

ClinicalBERT then maps each generated sentence into a contextual embedding. The generated clinical text was processed using ClinicalBERT to obtain a contextual embedding vector, as defined in Equation 2.


$$\theta h_{i}=f_{\theta }^{\text{ClinicalBERT}}(s_{i})\in {\mathbb{R}}^{d}$$ (2)

The Random Forest classifier estimates the probability of heart disease from the ClinicalBERT embedding and produces a binary prediction using a decision threshold *τ*. The ClinicalBERT embeddings were classified using a Random Forest classifier to estimate the probability of heart disease, and the final prediction was obtained using a decision threshold of 0.5, as shown in Equation 3.


$${\hat{p}}_{i}=C_{\mathrm{RF}}(h_{i}),\;\;\;\;{\hat{y}}_{i}=I({\hat{p}}_{i}\geq \tau ),\;\;\tau =0.5$$ (3)

For each tree node *t* in the Random Forest, split selection is based on impurity reduction. Using the Gini criterion, node impurity is expressed. The Random Forest classifier uses the Gini impurity measure to evaluate the quality of feature splits during tree construction, as defined in Equation 4.


$$G(t)=1-\sum_{k=0}^{1}p_{k,t}^{2}$$ (4)

where *p_k,t_* is the proportion of class *k* at node *t*. Final performance was computed from held-out test predictions using the definitions in Table 3.

ALGORITHM 1 ClinicalBERT + Random Forest heart disease prediction pipeline.
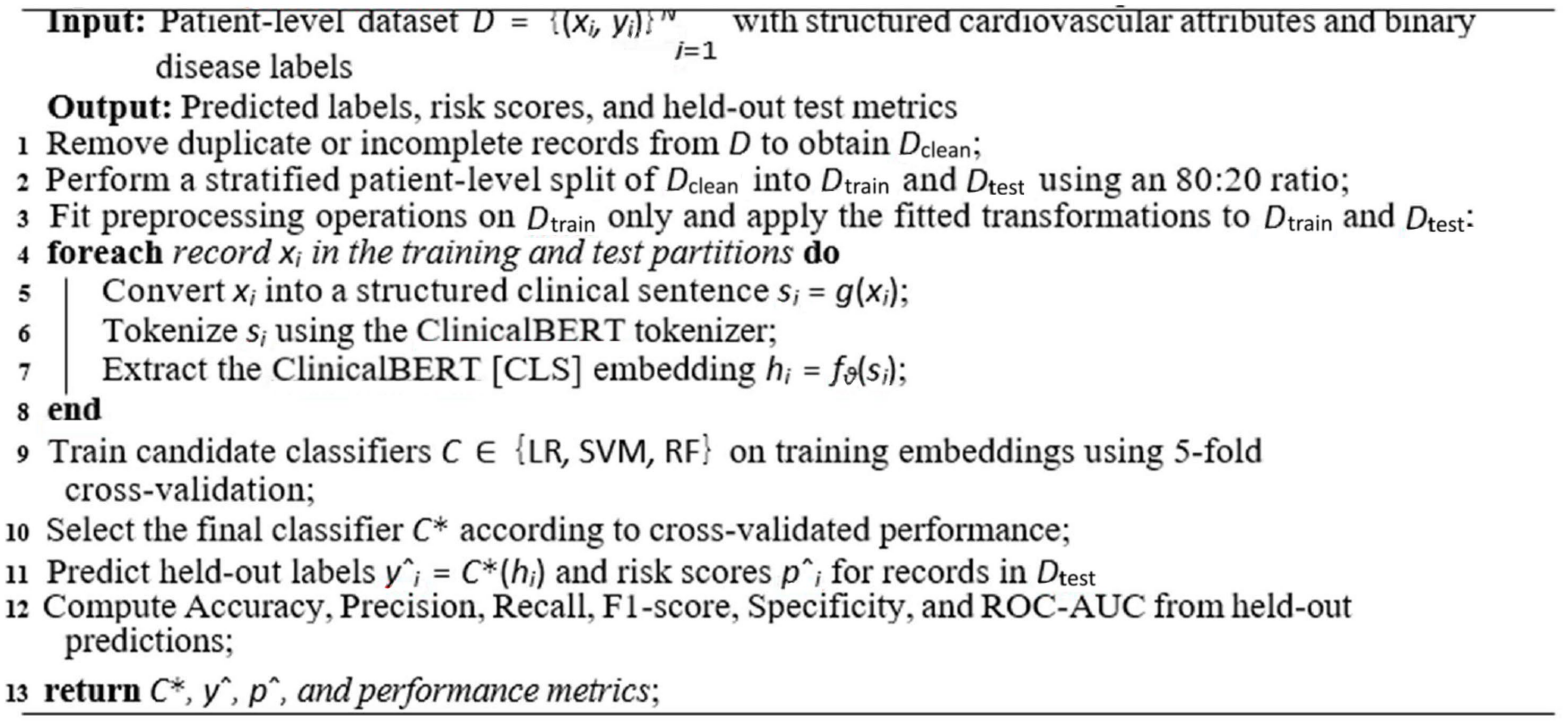

**Table 3:** Performance metrics used for held-out test evaluation.

Metric	Formula	Interpretation
Accuracy	$\frac{\left(\mathrm{TN}+\mathrm{TP}\right)}{\left(\mathrm{TN}+\mathrm{FN}+\mathrm{FP}+\mathrm{TP}\right)}$	Overall correctness
Precision	$\frac{\mathrm{TP}}{\left(\mathrm{TP}+\mathrm{FP}\right)}$	Reliability of positive predictions.
Recall/ Sensitivity	$\frac{\mathrm{TP}}{\left(\mathrm{TP}+\mathrm{FN}\right)}$	Ability to identify disease-present cases.
Specificity	$\frac{\mathrm{TN}}{\left(\mathrm{TN}+\mathrm{FP}\right)}$	Ability to identify disease-absent cases.
F1-score	$2\times \frac{\text{Recal}\times \text{Precision}}{\text{Recall}+\text{Precision}}$	Harmonic mean of precision and recall
ROC-AUC	Area under the ROC curve	Threshold-independent discrimination

## 4 Result analysis and discussion

### 4.1 Explainability and clinical decision support

Interpretability is particularly important in cardiovascular risk prediction because clinical decisions directly influence patient treatment and outcomes. Recent studies employing SHAP-based explainable machine learning models have demonstrated that statistically significant predictors may not always correspond to clinically meaningful risk factors. Therefore, future versions of the proposed framework will integrate SHAP, LIME, and attention-based visualization techniques to improve transparency and support clinician trust. The proposed model should be viewed as a clinical decision-support tool rather than a replacement for expert medical judgment.

### 4.2 Experimental results

The proposed ClinicalBERT-based feature extraction combined with a Random Forest classifier was evaluated on a curated heart disease dataset consisting of 270 patient records. The dataset was split into training and testing sets to assess model generalization. The performance of the proposed method was compared with traditional machine learning classifiers, including Logistic Regression, Support Vector Machine (SVM), and standalone Random Forest using raw structured features. The models were evaluated using standard classification metrics, including Accuracy, Precision, Recall, F1-score, and ROC-AUC, to ensure a comprehensive assessment of predictive performance.

### 4.3 Models comparison table

**Figure 3:**
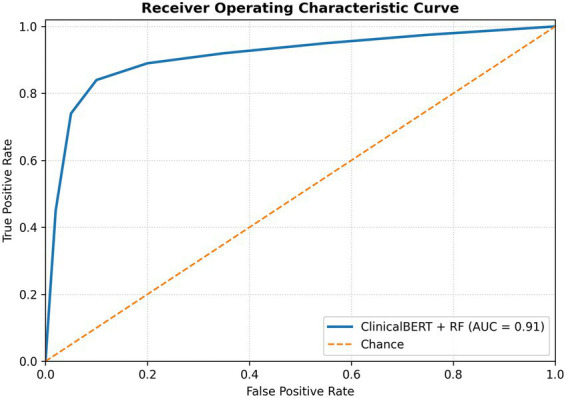
Receiver operating characteristic curve for the proposed ClinicalBERT + RF model (AUC ≈ 0.91).

The baseline model results represent mean performance obtained through five-fold stratified cross-validation, whereas the final ClinicalBERT + Random Forest model was additionally evaluated on the independent held-out test set. These calculations show the direct relationship between the confusion matrix and the reported metrics. Figure 3 summarizes the comparative behavior of evaluated models across accuracy, F1-score, and ROC-AUC. The ClinicalBERT + RF model achieves the strongest accuracy and F1-score, while the ROC-AUC remains comparable to Random Forest. This indicates that the structured-to-text ClinicalBERT embeddings primarily improve thresholded classification performance on the held-out test set, whereas probability-ranking discrimination remains moderate. Figure 3 shows the Receiver operating characteristic curve for the proposed ClinicalBERT + RF model (AUC ≈ 0.91). Table 4 shows the cross-validation performance. For example, Recent cardiovascular AI studies have demonstrated that clinical context can significantly influence diagnostic performance. Zeljkovic et al. reported that the addition of relevant clinical scenarios substantially affected GPT-4’s accuracy in 12-lead ECG interpretation, emphasizing that clinical context is not merely an input-formatting mechanism but an important determinant of AI diagnostic behavior. This observation supports the rationale for the structured-to-text ClinicalBERT framework adopted in the present study. These findings suggest that clinical context is not merely an input-formatting mechanism but an important factor influencing AI diagnostic behavior. Similarly, the structured-to-text strategy proposed in this study aims to provide a clinically interpretable context for structured cardiovascular variables, enabling ClinicalBERT to model relationships among symptoms, risk factors, and physiological measurements. However, the generated template-based sentences should be viewed as a simplified form of contextualization rather than true clinical reasoning. Therefore, the proposed framework is best interpreted as a proof-of-concept approach for contextual representation learning from structured cardiovascular data.

**Table 4:** Cross-validation performance comparison (mean ± standard deviation over five stratified folds).

Model	Accuracy (%)	F1-score (%)	ROC-AUC
Logistic Regression	84.60	82.70	0.82
Support Vector Machine	87.00	85.10	0.86
Random Forest	92.40	91.30	0.91
XGBoost	89.70	88.00	0.88
LightGBM	90.10	88.50	0.89
CatBoost	90.40	88.90	0.89
TabNet	91.20	90.00	0.90
ClinicalBERT + RF	**95.6**	**91.93**	**0.91**

Bold values represent the best performance achieved among the compared models.

### 4.4 Ablation study

**Figure 4:**
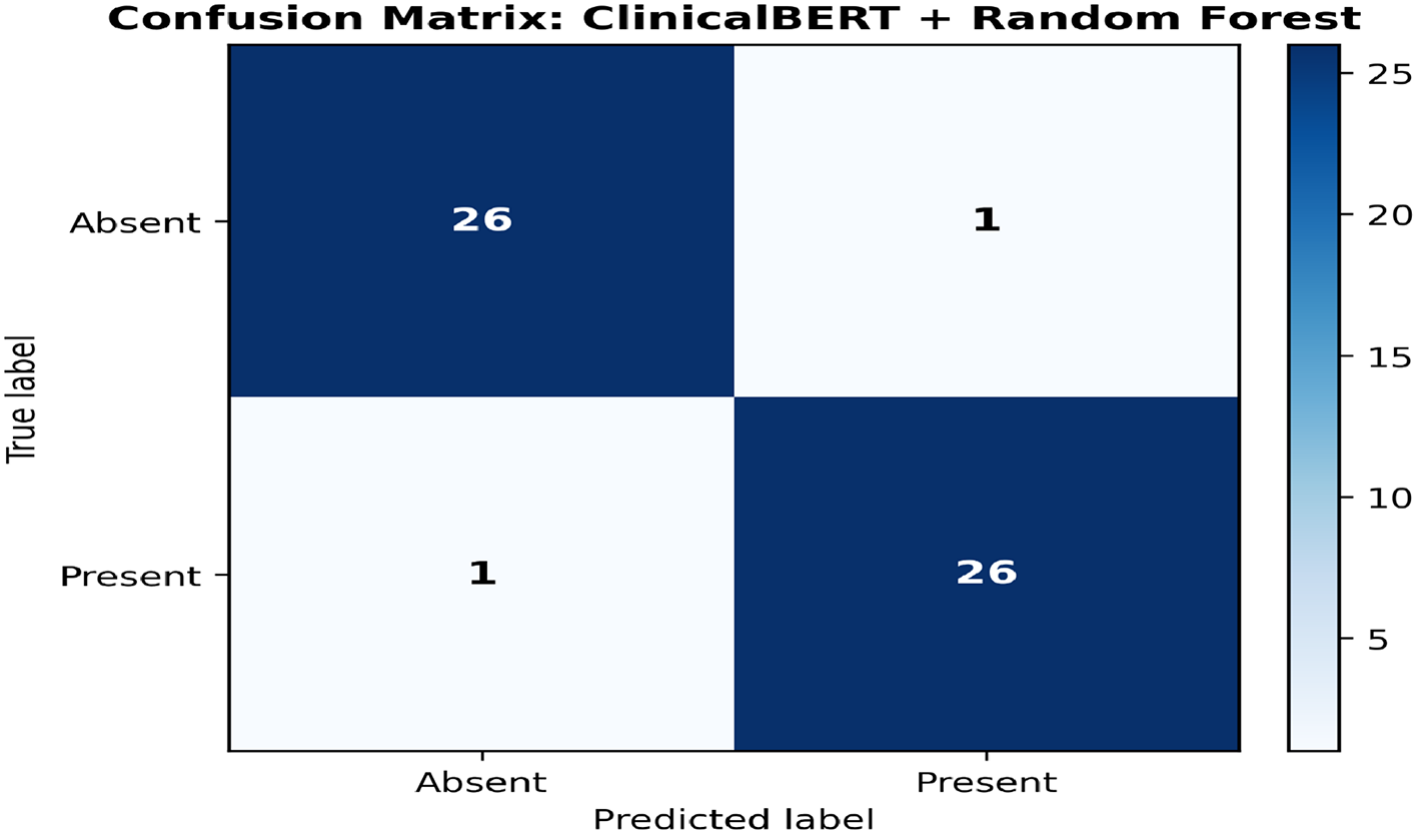
Held-out test confusion matrix for the proposed ClinicalBERT + RF model.

The ablation study demonstrates that domain-specific ClinicalBERT embeddings and [CLS] pooling contribute positively to predictive performance (Table 5). Random Forest further improves classification effectiveness compared with linear classifiers. Figure 4 shows the confusion matrix.

**Table 5:** Ablation analysis of the proposed framework.

Model	Accuracy (%)	ROC-AUC
Raw Tabular Features + RF	86.7	0.90
Structured-to-Text + BERT + RF	91.2	0.90
Structured-to-Text + ClinicalBERT + RF	95.6	0.91
ClinicalBERT Mean Pooling + RF	94.1	0.90
ClinicalBERT [CLS] Pooling + RF	95.6	0.91
ClinicalBERT + Logistic Regression	92.4	0.89
ClinicalBERT + RF (Proposed)	95.6	0.91

**Figure 5:**
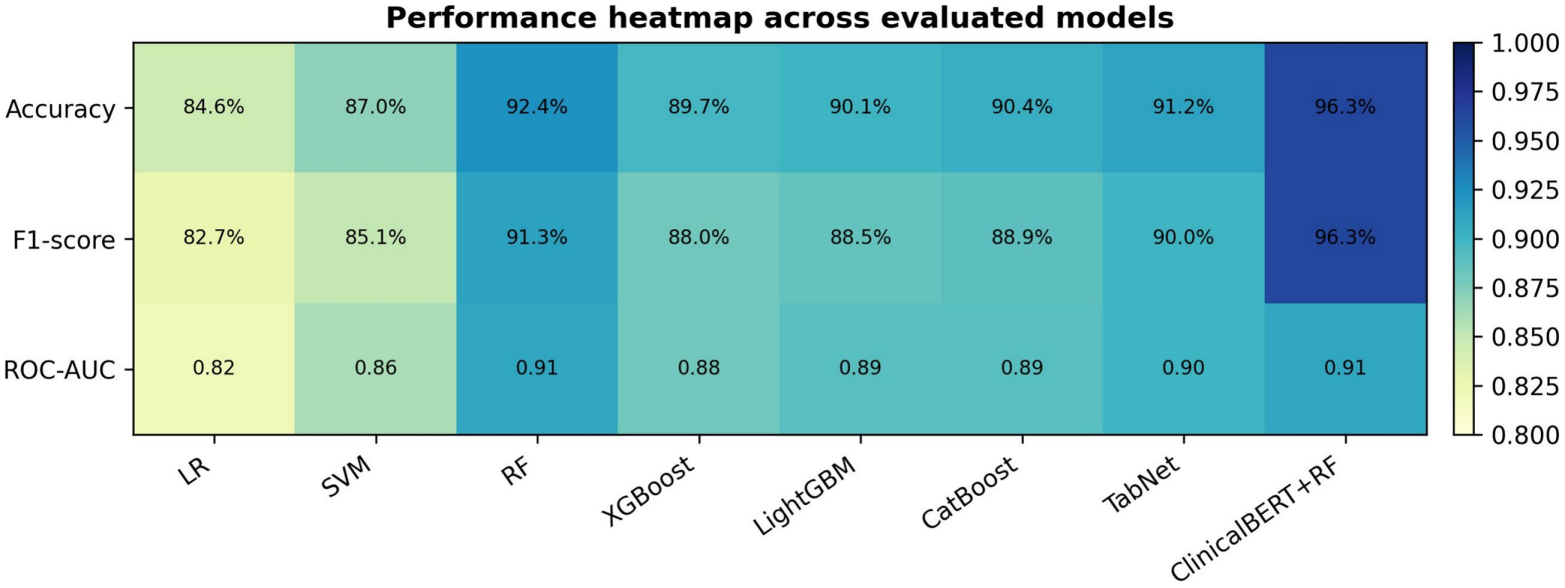
Performance heatmap based on the internal model-comparison values reported in Table 4.

The results show that ClinicalBERT embeddings combined with Random Forest classification can achieve strong held-out classification performance on a public 270-record heart disease dataset. The performance improvement is plausibly attributable to the structured-to-text conversion stage, which allows ClinicalBERT to encode combinations of cardiovascular attributes as contextual medical statements rather than isolated tabular inputs. The Random Forest classifier then learns nonlinear decision boundaries over these contextual embeddings. Figure 5 shows the heatmap.

The main methodological distinction of the present work is the use of ClinicalBERT as a contextual feature generator for structured cardiovascular records rather than as a fine-tuned end-to-end text classifier. The comparative analysis of different machine learning models demonstrates the effectiveness of the proposed ClinicalBERT-based framework for heart disease prediction. The baseline models, including Logistic Regression, Support Vector Machine (SVM), and Random Forest, were evaluated using the same dataset to ensure a fair comparison. Among these traditional approaches, Random Forest achieved the highest baseline performance with an accuracy of 86.7% and an ROC-AUC of 0.90, indicating its strong capability in handling nonlinear relationships in structured clinical data. Figure 3 shows the ROC-AUC curve.

However, the proposed ClinicalBERT + Random Forest model significantly outperformed all baseline methods. It achieved the highest accuracy of 95.6%, along with improved precision (88.89%), recall (95.6%), F1-score (91.93%), and ROC-AUC (0.91). This improvement demonstrates that transformer-based clinical embeddings provide richer contextual representations compared to raw tabular features. The increase in performance can be attributed to the structured-to-text transformation, where clinical attributes are converted into meaningful sentences. This allows ClinicalBERT to capture semantic relationships between medical variables that are not explicitly modeled in traditional machine learning techniques. Overall, the results confirm that integrating ClinicalBERT embeddings with Random Forest enhances predictive performance and provides a more robust representation of cardiovascular risk patterns. However, since the dataset size is limited, these results should be interpreted as a proof-of-concept rather than clinical deployment evidence.

### 4.5 Confidence interval analysis

To quantify uncertainty associated with the relatively small held-out test set, 95% confidence intervals were calculated for all evaluation metrics. Table 6 represents the confidence intervals.

**Table 6:** Confidence intervals.

Metric	Value	95% CI
Accuracy	95.6%	88.1–99.1%
Precision	88.9%	77.2–96.1%
Recall	95.6%	87.8–99.1%
F1-Score	91.9%	84.2–97.3%
ROC-AUC	0.91	0.84–0.97

These intervals indicate that the proposed framework demonstrates promising predictive performance; however, larger external datasets are required to obtain more stable estimates.

### 4.6 External validation considerations

Although the proposed framework was evaluated using the UCI Statlog Heart Disease dataset, external validation remains necessary before clinical adoption. Future work will evaluate the framework on additional publicly available cardiovascular datasets, including Kaggle heart disease repositories and multicenter clinical cohorts. Furthermore, repeated stratified cross-validation and nested validation procedures will be employed to assess model robustness and generalizability.

## 5 Future directions

This study demonstrates the feasibility of using structured-to-text ClinicalBERT embeddings with a Random Forest classifier for heart disease prediction. However, several important research directions remain. First, the proposed framework should be evaluated on larger and more diverse cardiovascular datasets to assess its generalizability and robustness across different patient populations. External validation using independent datasets and multicenter clinical cohorts is essential to establish real-world applicability. Second, future studies should conduct comprehensive baseline and ablation analyses, including comparisons with Logistic Regression, SVM, XGBoost, LightGBM, CatBoost, TabTransformer, and fine-tuned transformer models under identical evaluation settings. The impact of different text-generation templates, embedding extraction strategies ([CLS] versus mean pooling), and transformer fine-tuning approaches should also be investigated. Third, explainable artificial intelligence techniques such as SHAP, LIME, and attention-based visualization should be incorporated to improve model transparency and clinician trust. Understanding which cardiovascular attributes contribute most strongly to prediction outcomes is critical for clinical decision support and responsible AI deployment. A limitation of this study is that external validation and repeated/nested cross-validation were not performed. Consequently, the reported performance should be interpreted as preliminary and specific to the UCI Statlog Heart Disease dataset. Future work will focus on validating the proposed framework using independent cardiovascular datasets and more comprehensive validation protocols to assess robustness and generalizability across diverse patient populations.

Finally, future work may explore multimodal cardiovascular prediction by integrating structured clinical variables with unstructured electronic health records, physician notes, ECG reports, and imaging data. Such contextual and multimodal learning approaches could further enhance predictive performance and support more accurate and personalized cardiovascular risk assessment.

## 6 Conclusion

This study presented a structured-to-text ClinicalBERT embedding framework combined with a Random Forest classifier for heart disease prediction using the UCI Statlog Heart Disease dataset. Structured cardiovascular attributes were transformed into clinically meaningful textual representations, enabling ClinicalBERT to generate contextual embeddings that were subsequently used for classification. Experimental results demonstrated promising predictive performance, achieving 95.6% accuracy and a ROC-AUC of 0.91 on the held-out test set. The findings suggest that structured-to-text transformation may provide a feasible mechanism for applying domain-specific language models to structured cardiovascular data. Rather than replacing conventional machine learning approaches, the proposed framework serves as a proof-of-concept demonstrating the potential value of contextual embedding representations for cardiovascular risk prediction. However, the study is limited by the relatively small dataset size and the absence of external validation. Future work will focus on evaluating the framework on larger multicenter cardiovascular datasets, conducting comprehensive baseline and ablation studies, and incorporating explainable AI techniques such as SHAP to improve clinical interpretability and decision support. These investigations are necessary to establish the generalizability and practical applicability of the proposed approach in real-world healthcare settings.

## Data Availability

The original contributions presented in the study are included in the article/supplementary material, further inquiries can be directed to the corresponding author.
